# Volatile Compounds in Norway Spruce (*Picea abies*) Significantly Vary with Season

**DOI:** 10.3390/plants12010188

**Published:** 2023-01-02

**Authors:** Katja Schoss, Nina Kočevar Glavač, Samo Kreft

**Affiliations:** Department of Pharmaceutical Biology, Faculty of Pharmacy, University of Ljubljana, Aškerčeva 7, 1000 Ljubljana, Slovenia

**Keywords:** essential oil, hydrosol, chemical investigation, *Picea abies*, GC-MS

## Abstract

Norway spruce (*Picea abies*) is one of the most important commercial conifer species naturally distributed in Europe. In this paper, the composition and abundance of essential oil and hydrosol from the needles and branches of *P. abies* were investigated with an additional evaluation of changes related to different times of the year, annual shoots and branches, and differences in composition under different microenvironments. Essential oils and hydrosols obtained via hydrodistillation were analyzed using gas chromatography–mass spectrometry (GC-MS), where 246 compounds in essential oil and 53 in hydrosols were identified. The relative amounts of monoterpenes, sesquiterpenes, and diterpenes in essential oil changed significantly during the year, with the highest peak of monoterpenes observed in April (72%), the highest abundance of sesquiterpenes observed in August (21%), and the highest abundance of diterpenes observed in June (27%). The individual compound with the highest variation was manool, with variation from 1.5% (April) to 18.7% (June). Our results also indicate that the essential oil with the lowest allergenic potential (lowest quantity of limonene and linalool) was obtained in late spring or summer. Location had no significant influence on composition, while the method of collection for distillation (whole branch or annual shoots) had a minor influence on the composition. All nine main compounds identified in the hydrosol samples were oxygenated monoterpenes. The composition of *P. abies* hydrosol was also significantly affected by season. The method of preparing the branches for distillation did not affect the composition of *P. abies* hydrosol, while the location had a minor effect on composition.

## 1. Introduction

Since ancient times, plants have been a source of bioactive compounds that humans use for beneficial health effects and as a resource for drug development. At present, there is significant interest in the use of natural plant-based bioactive compounds, especially when the material is sustainably accessible and can work within the circular economy. An important example of such a material is the branches of *Picea abies* (L.) H. Karst (Pinaceae), commonly known as European or Norway spruce, which is a tall essential-oil-bearing evergreen conifer used mostly for its wood [1,2]. *P. abies* accumulates volatile compounds in all parts of the plant, with the highest abundance in needles [3], which is also the least used part in the wood industry and the part of the plant with the largest volume [4]. Thus, in terms of sustainability, it is logical to exploit these needles for beneficial purposes.

Essential oils or volatile compounds (terpenes, terpenoids, and phenylpropanoids) are formed in aromatic plants as secondary metabolites [5] and play an important role in plant defense. Essential oils are typically produced through a distillation process where two products are obtained: an essential oil and a hydrosol. In European Pharmacopoeia, essential oils are described as odorous products, usually of complex composition, which are obtained from a botanically defined plant raw material via steam distillation, dry distillation, or a suitable mechanical process without heating [6]. Hydrosols, also known as hydrolats or aromatic waters, are not yet described in the European Pharmacopoeia. Hydrosols are generally known as the aqueous phase obtained from distillation that separates them from the essential oil phase and are highly diluted, acidic, and scented solutions containing variable amounts of essential oil along with other volatile water-soluble compounds (up to approximately 0.1%) [7,8]. While the scientific literature on essential oils is relatively abundant, much less is known about hydrosols. The compositional profiles of hydrosols may or may not qualitatively overlap with those of the corresponding essential oils, although hydrosol profiles typically differ significantly in quantitative terms [7].

Essential oils are known for their variety of activities. For example, conifer essential oils are known for their antibacterial, antifungal, insect larvicidal, antioxidant, anti-inflammatory, cytotoxic, and antiproliferative activities [9,10,11]. The investigation of hydrosol biological activity has focused mainly on their antimicrobial, antioxidant, and anti-inflammatory activities [7,12].

The volatile compounds of coniferous species are monoterpenes, sesquiterpenes, and diterpenes [10]; however, the qualitative and quantitative composition of such compounds depends on several factors, such as the anatomical part of the tree (needles, branches, or cones), genetic factors, and the health condition of the plant, as well as the environment, location, light quality, seasonal variations, and isolation and determination techniques used for analysis [5,10,11,13,14,15]. Thus, the composition of essential oils and hydrosols varies even within the same species. Since biological activity is dependent on chemical composition, such activity is similarly subject to variation. Although hydrosols are produced through the same distillation process as essential oils, their analyses have been the subject of a limited number of publications, despite their current popularity in the aromatherapy, food, and cosmetic industries [8].

The aim of this study was to investigate the composition and abundance of essential oils and hydrosols from the needles of *P. abies* and evaluate changes during different times of the year, as well as comparing the composition of annual shoots and branches and differences in composition in different microenvironments. The effects of seasonal variations on the chemical and biological characteristics of some essential oils of the Pinaceae family were previously reported in the literature [3,16,17]. However, no detailed reports are available on the seasonal chemical composition of the essential oil of *P. abies*. To the best of our knowledge, this is also the first publication on the composition of *P. abies* hydrosol.

## 2. Results and Discussion

### 2.1. Essential Oil Yield

The yield of essential oil in the annual shoots and branches of *P. abies* ranged from 0.02 to 0.34% (mL relative to fresh material) (Table 1), which means that 10 to 170 µL of essential oil was obtained during the distillation process from 50 g of fresh material. In the whole branch samples, the minimum oil yield of 10 µL was found in location 4, while the maximum yield (170 µL) was found in location 1. The yield was significantly (*p* = 0.001) influenced by the time of harvesting, with the highest in the fall, the lowest in both spring harvests, and another slight increase over the summer. The location and difference between the whole branch and shoots had no statistical effect on the amount of essential oil. The yield results are in agreement with those of Baath et al. [17], who observed an average yield from fresh needles of 0.07%. In contrast, our results are lower than the results from Radulescu et al. [11] and Visan et al. [10], who recorded an average abundance of essential oils of 1.01% and 1.02%, respectively. However, the yield in those studies was determined for dried plant material.

### 2.2. Essential Oil Composition

A total of 246 different compounds were found in 32 samples, as presented in Appendix A. The abundance of all compounds is expressed as a percentage of the relative peak areas (percentage of the area of the chromatographic peak of an individual compound compared to the sum of the areas of all chromatographic peaks). A total of 17 compounds had an average abundance greater than 1.5% and are presented in Table 2. Only 8 compounds were detected in all 32 samples: limonene, β-pinene, borneol, camphene, abienol, α-pinene, T-muurolol, and α-humulene (in Table 2 marked in bold). The compound with the highest average relative peak area percentage detected in the samples, despite not being present in all samples, was manool, with a maximum abundance of 40.88% and an average abundance of 10.96%. In some studies, no diterpenes were detected in essential oils, as GC temperatures were set below 250 °C [9,18], while in studies using GC methods above 250 °C, manool was detected in greater quantities (Visan et al. [10]: 9.4%; Radulescu et al. [11]: 2.6–6.2%). The same applies to α-cadinol, which was observed at a maximum of 6.41% in our study and a maximum of 16.56% in the study by Garzola et al. [9]. In the study by Kartnig et al. [18], this compound was not observed, and in Visan et al. [10] and Radulescu et al. [11], this compound was present in 3.8% and 11.2–25.3%, respectively.

The main compound classes in the studied essential oils were monoterpenes (monoterpene hydrocarbons: 22.89%; oxygenated monoterpenes: 19.34%), followed by sesquiterpenes (sesquiterpene hydrocarbons: 6.61%; oxygenated sesquiterpenes: 8.79%) and diterpenes (diterpene alcohols: 14.79%).

These results are in partial agreement with four other studies. Baath et al. [17], who investigated the essential oil composition of 16 different conifer species, including *P. abies*, reported α-pinene, camphene, limonene, and bornyl acetate to be the four predominant compounds; in our study, these compounds ranked ninth, seventh, third, and second. In a study by Garzoli et al. [9], the main components analyzed by GC-MS were β-pinene (44.7%), α-pinene (20.2%), limonene (14.2%), and camphene (7.2%); in our study, these compounds ranked fifth, ninth, third, and seventh, respectively. When analyzed by GC-headspace [9], the results were as follows: β-pinene (43.8%), α-pinene (34.5%), camphene (10.5%), and limonene (8.0%). In a study by Visan et al. [10], the compounds with the highest abundance were limonene (21.1%), α-pinene (11.6%), bornyl acetate (11.08%), camphene (10.7%), and manool (9.4%). In a study by Radulescu [11], the composition of essential oil was also evaluated considering geographical aspects. Plants were collected from different parts of Romania, and the geographical influence was significant, but the highest- abundance compounds remained similar throughout (α-cadinol, 11.2–25.3%; bornyl acetate, 11.8–15.0%; camphene, 3.8–14.1%; and limonene, 6.3–13.0%. These compounds were ranked in our study as twentieth, second, seventh, and third, respectively (see Appendix A)).

Most compounds present in higher percentages were previously studied for their potential biological or therapeutic activities, which suggests possible usages of *P. abies* essential oil in conventional and complementary medicine, such as aromatherapy. Manool possesses antigenotoxic, anticarcinogenic, and anti-inflammatory potential [19], while an anti-melanoma effect was shown in vivo in mice [20]. Bornyl acetate possesses anti-inflammatory [21] properties; limonene possesses antihyperalgesic [22,23], antidiabetic [24], anti-inflammatory [25], and antioxidative [25] potential; α-cadinol has antifungal [26] properties; β-pinene antimicrobial [27] has antioxidative properties [28] and, in combination with linalool, antidepressant properties [29]; borneol has antiglycemical [30], antihyperlipidemic [30], antioxidative [30], and antinociceptive [31] properties; camphene has antitumor [32], antioxidative [33], and hypolipidemic [34] properties; abienol has antifungal, antimicrobial, and antineoplastic properties [35]; α-pinene has antimicrobial [27,36], antioxidative [28], anti-inflammatory [37], gastroprotective [38], and antinociceptive properties [39]; myrcene has antioxidative [40] and anti-inflammatory properties [41]; (E)-caryophyllene has anti-inflammatory [42], anticonvulsant [43], and antinociceptive properties [44]; T-muurolol has antifungal properties [26]; δ-cadinene has anticancer [45] and acaricidal properties [46]; α-humulene has anti-inflammatory properties [47]; and camphor has anti-inflammatory [48], eucalyptol antibacterial [49], anti-inflammatory [50,51], and antioxidative [51] properties.

*P. abies* essential oil is also widely used as a cosmetic ingredient, where it is desirable for the oil to contain monoterpene abundance that is as low as possible, since monoterpenes are generally responsible for allergic reactions. In *P. abies* essential oil, citronellol and limonene were recognized as typical allergens [52], and their use in cosmetic products is regulated [53]. Limonene was also found among the main compounds in the studied samples (average yield of 6.86%). In addition, some monoterpenes are highly prone to oxidation, such as α-pinene and β-pinene (in this study, they were detected at a yield of 3.25% and 5.59%, respectively), and air-oxidized products were shown to be potent skin allergens [54].

### 2.3. Variation of Essential Oil Composition

Comparing the composition of essential oils obtained from plant materials at different times of the year, we found that shares of monoterpenes, sesquiterpenes, and diterpenes greatly varied during the year, as shown in Table 2. The abundance of both monoterpene hydrocarbons and oxygenated monoterpenes was the highest in April (38% and 34%, respectively) and lowest in June (4% and 9%, respectively). The abundance of total sesquiterpenes was highest during August (20.63%) and lowest during April (6.88%), and the abundance of diterpenes was highest during June (27.17%) and lowest during April (3.83%). The individual compounds that varied most significantly (more than 5%) were manool (18.73% in June and 1.51% in April), bornyl acetate (17.64% in April and 4.19% in June), limonene (9.05% in November and 1.20% in June), α-cadinol (10.55% in August and 2.44% in April), and β-pinene (10.56% in April and 1.15% in June).

Organoleptically, in terms of odor, the most desirable component of *P. abies* essential oil is bornyl acetate, which provides the typical pleasant smell of needles, while the least desirable is myrcene, which produces an unpleasant smell of plastic. Both compounds were highest in early spring, fell sharply over the course of spring and then slowly increased again through summer and autumn.

The allergens limonene and linalool varied in the same way, i.e., with a peak in April, a sharp decrease in June, and a subsequent increase from August to November, while a minor amount of citronellol (0.01%) was detected only in April. Our results indicate that the essential oil with the least allergenic potential was obtained in late spring or summer.

A study by Kamaitytė-Bukelskienė et al. [3] studied the content variation of α-pinene and β-pinene from May to September and observed the highest abundance in May, which corresponds well with our results.

We conclude that season has the strongest influence on the composition of *P. abies* essential oil, generally providing the largest change (increase or decrease) from April to June. Of the 17 compounds with the highest abundance, differences between seasons were significant (*p* < 0.05) for 13 compounds (Table 2). The abundance of all seven sesquiterpenes and diterpenes increased sharply in spring, while at the same time, the abundance of all ten monoterpenes dropped. The largest change was observed for the diterpene manool and the monoterpenes limonene and eucalyptol. Manool increased by more than a factor of 10, and limonene and eucalyptol decreased by a factor of more than 10.

The location had no significant influence on the composition of spruce essential oil, which was expected since the distances between the locations were smaller than 20 km. Nevertheless, the sampling on four locations additionally supported the dependence on seasonal variation.

The method of branch collection had little effect on the composition of *P. abies* essential oil (Table 2). The differences between the essential oil in annual shoots and whole branches were significant for none of the 17 compounds with the highest abundance. The largest difference was observed in the abundance of β-pinene, which was 2× more common in the essential oil from whole branches than in the essential oil from annual shoots. Conversely, the abundance of bornyl acetate in essential oil from whole branches was 30% lower than that in essential oil from annual shoots. Since bornyl acetate is a desired component of *P. abies* essential oil and β-pinene is not, better quality essential oil can be obtained using only annual shoots, but this requires more manual labor.

The method used for preparing the branches before distillation (the annual shoots of the third harvesting term were prepared by grinding in addition to the standard method) did not significantly affect the composition of the essential oil (data not shown), but the yield was 2.5× higher in the distillation of grinded samples (*p* = 0.017) (see Table 1).

Based on the data from this study, we conclude that the composition of *P. abies* essential oil varies greatly with season but not with short-distance geographic origin or the preparation of distilled material.

### 2.4. Hydrosol Composition

Although hydrosols are produced through the same distillation process as essential oils, their analyses have been subject to a limited number of publications. To the best of our knowledge, this is the first publication on the composition of *P. abies* hydrosol. Similar to essential oils, it is believed that the composition of hydrosols produced from plant parts of the same species differs when collected in different countries, different geographical locations within a country, different seasons, or at different plant growth stages, consequently altering their biological activities [8]. The applied analysis technique, or the preparation of the sample for analysis (extraction with organic solvent or direct analysis), also has an important influence on the results. As shown by Kokalj Ladan et al. [55], direct hydrosol analysis should be used when the total composition of a hydrosol is the research focus because it allows for the identification of hydrophilic compounds that are not extracted into the organic solvent phase, and true ratios among compounds are obtained. However, it has to be emphasized that direct analysis has poorer repeatability. Based on the above-mentioned advantages of direct hydrosol analysis this approach was selected for compositional analysis in this study.

The first observation based on the results shown in Table 2 and Table 3 shows a large difference in the qualitative and quantitative composition of the essential oil and hydrosol. In the 32 hydrosol samples of *P. abies*, a total of 53 different compounds were found. The abundances of all compounds are expressed here as a percentage of the relative peak areas (percentage of the area of the chromatographic peak of an individual compound compared to the sum of the areas of all chromatographic peaks). This percentage, at the same time, corresponds to the weight percentage representation of this compound in relation to the non-aqueous (terpene) part of the hydrosol. Nine compounds had abundance greater than 1.2% (Table 3).

All of these compounds were oxygenated monoterpenes, with the exception of hexenol and hexenal, which were likely artefacts. The dominant abundance of oxygenated monoterpenes in hydrosols is not unusual and also appeared in other studies on the composition of hydrosols of conifers [56,57]. Only one compound (camphene hydrate) was detected in all 32 samples.

Among the most abundant compounds in hydrosols, only four were also the most abundant in the essential oil: borneol, camphor, camphene hydrate, and eucalyptol. All the most commonly represented compounds in hydrosols had a logP value (octanol/water) of less than 3, indicating that they are hydrophilic to moderately lipophilic. Compounds that were strongly represented in the essential oil but not in the hydrosol all had a logP greater than 4. Bornyl acetate, which has a logP of 3.6 (i.e., between 3 and 4), was present in the hydrosol with less than an average of 1%, ranked 14th and present in the essential oil with an average of 32% (ranked second). It should also be taken noted that the solubility of a compound in water at a neutral pH may be different than that at a pH between 3.5 and 4, which is the typical pH of a hydrosol [56,57].

The composition of *P. abies* hydrosol was, as is common for essential oils, significantly affected by the season. Of the nine compounds with the highest abundance, differences between seasons were significant for five compounds. However, the pattern of changes was not as uniform as that with essential oils. Only with eucalyptol and camphor did we observe the same pattern as that seen in essential oils, i.e., with the maximum abundance appearing in early spring (April), followed by a sharp fall in June and then a slow increase again by November.

The method of preparing the branches for distillation did not affect the composition (Table 3). The differences between collections were significant for none of the nine compounds with the highest abundance.

The location, moreover, had little effect on the composition of spruce hydrosol. The differences between sites were significant (data not shown) for none of the nine compounds with the highest abundance.

## 3. Materials and Methods

### 3.1. Plant Material and Sample Preparation

Branches of *Picea abies* (L.) Karst. were collected at four locations in the municipality of Loški Potok, Slovenia, in the settlements of Blošček (location 1; N: 45°43′59″, E: 14°33′13″; 809 m), Bela Voda (location 2; S: 45°41′20″, E: 14°37′09″; 924 m), Hrib (location 3; S: 45°41′49″, E: 14°37′16″; 868 m), and Lazec (location 4; S: 45°39′28″, E: 14°37′36″; 802 m). Plants were identified by Samo Kreft, and the voucher specimen of the plant was deposited in the Herbarium of the Department of Pharmaceutical Biology, Faculty of Pharmacy, University of Ljubljana, Slovenia (voucher No. BF00159/2017). Branches were collected four times (5 November 2017; 4 April 2018; 28 May 2018; and 13 August 2018) from growing (uncut) trees, up to a height of 5 m and cut at a branch thickness of approximately 1 cm.

Fresh whole branch samples, including annual shoots, were collected from every location and cut into 5 to 10 cm long pieces. Additionally, annual shoots (approximately 10 cm long unbranched thin twigs with needles) were collected separately.

In total, 50 g of each branch sample was put into a 1000 mL round flask with 500 mL of demineralized water and distilled for 4 h using a Clevenger-type apparatus according to general instructions of European Pharmacopoeia, “2.8.12. Determination of essential oils in herbal drugs” (6). No solvent was used during hydro-distillation. All samples (hydrosols and essential oils) were stored after distillation until analysis in glass tubes sealed with a glass stopper and frozen at −20 °C.

For additional comparison, distillation from ground annual shoots obtained from all locations at one time period (28 May 2018) was also performed (the particle size of the branches and needles was approximately 3 mm).

### 3.2. Gas Chromatography Coupled with Mass Spectrometry (GC-MS)

Essential oil (diluted in n-hexane (Suprasolv, Merck, Germany) (1 mg/mL)) and hydrosol (unprocessed) samples were analyzed on a Shimadzu GC-MS system GCMS-QP2010 Ultra equipped with a MS detector and Rxi-5Sil MS (Restek, Bellefonte, PA, USA) capillary column (30 m × 0.25 mm, film thickness 0.25 μm). The injector and ion source temperatures were set to 250 and 200 °C, respectively. The column temperature was programmed from 50 to 250 °C at a rate of 3 °C/min, and the lower and upper temperatures were held for 5 min. Helium (99.99%) was used as carrier gas at a flow rate of 1 mL/min. A sample of 1.0 μL was injected with an autosampler using the split mode with a split ratio of 1:100. For MS detection, an electron ionization mode with an ionization energy of 70 eV was used. The MS transfer line temperature was set to 250 °C. The m/z range was from 40 to 400 with a scanning frequency of 5 Hz.

Identification of the compounds was done based on a comparison of their mass spectra and retention indices with those of the synthetic compounds in the spectral library of the National Institute of Standards and Technology (NIST11), as well as the Flavors and Fragrances of Natural and Synthetic Compounds spectral library (FFNSC2). The linear retention indices were determined in relation to a homologous series of n-alkanes (C6-C24). Component relative concentrations were calculated from the GC peaks without using correction factors.

### 3.3. Statistical Analysis

All of the GC-MS analyses were conducted in duplicates. Statistical analysis of the data was performed by calculating the average, standard deviation (STD) and analysis of variance (ANOVA) using the SPSS 26 software. No variable transformation was applied. A probability value at *p* ≤ 0.05 was considered statistically significant.

## 4. Conclusions

The main compounds of *P. abies* essential oil analyzed in the present study were manool, bornyl acetate, limonene, α-cadinol, and β-pinene, while those of hydrosol were borneol, camphor, and α-terpineol. The comparison of essential oil composition between seasons showed significant variation in the abundance of monoterpenes and sesquiterpenes, as well as significant variation of monoterpenes in hydrosols.

Since the essential oil and hydrosol of *P. abies* have a wide option of possible applications in the pharmaceutical, cosmetic, and food industries, it is of great importance to define the composition of *P. abies* and consider variations in composition during the year. The information observed on seasonal variation may be useful in selecting the best season for essential oil or hydrosol distillation, as well as for choosing the product with the most desirable properties, such as the essential oil with the fewest allergens.

## Figures and Tables

**Table 1:** Yield of essential oil (%) obtained from 50 g of each sample.

Date	Preparation of Branches	Location 1	Location 2	Location 3	Location 4	Average
5 November 2017	Annual shoots	0.22	0.08	0.06	0.12	0.12
	Whole branch	0.34	0.12	0.08	0.08	0.16
4 April 2018	Annual shoots	0.03	0.10	0.03	0.03	0.05
	Whole branch	0.08	0.10	0.03	0.02	0.06
28 May 2018	Annual shoots	0.03	0.04	0.04	0.06	0.04
	Whole branch	0.05	0.06	0.09	0.06	0.07
	Annual shoots—ground	0.09	0.07	0.08	0.16	0.10
13 August 2018	Annual shoots	0.10	0.07	0.12	0.06	0.08
	Whole branch	0.07	0.05	0.08	0.08	0.07
Average	-	0.11	0.07	0.07	0.07	0.08 *

* without the results for “annual shoots—ground” samples.

**Table 2:** List of volatile compounds whose average abundance, according to their relative peak intensity (RPI) (Area %), was greater than 1.5% in 32 essential oil samples, with their minimum, maximum, and average abundance obtained at different season times and with different plant parts with included *p*-value as detected by gas chromatography-mass spectrometry. The compounds were identified based on their mass spectra and retention indices (RI; Db, database RI; Ms, measured RI).

Compound	RI		Minimum RPI (%)	Maximum RPI (%)	Average RPI (%)	Standard Deviation	No. of Samples without Compound	17 November		18 April		18 June		18 August		Seasons	Branch		Annual Shoots		Collection Method
*Chemical Group*	*Ms*	*Db*						Average	STD	Average.	STD	Average.	STD	Average	STD	*p*	Average	STD	Average	STD	*p*
**α-pinene**	930	933	0.10	9.18	3.25	3.12	0	5.47	3.30	4.84	2.74	0.56	0.48	2.14	2.51	0.00	3.87	3.35	2.64	2.84	0.27
*monoterpene hydrocarbon*																					
**camphene**	946	942	0.15	16.44	3.96	4.19	0	6.93	4.80	4.78	1.20	0.51	0.28	3.61	5.35	0.01	3.64	3.91	4.27	4.56	0.68
*monoterpene hydrocarbon*																					
**β-pinene**	974	978	0.11	28.51	5.59	6.63	0	7.68	5.99	10.56	9.02	1.15	1.34	2.99	3.49	0.01	7.77	7.97	3.41	4.14	0.06
*monoterpene hydrocarbon*																					
myrcene	987	991	0.00	10.95	3.23	3.09	2	4.04	2.90	5.04	2.22	0.61	0.49	3.23	4.04	0.02	3.20	2.92	3.26	3.34	0.96
*monoterpene hydrocarbon*																					
**limonene**	1027	1030	0.19	22.78	6.86	6.00	0	9.05	4.36	12.53	6.21	1.20	0.82	4.66	4.10	0.00	7.47	6.17	6.25	5.96	0.58
*monoterpene hydrocarbon*																					
eucalyptol	1030	1032	0.00	6.04	1.70	1.81	7	1.86	1.53	3.90	1.51	0.15	0.24	0.88	0.84	0.00	1.65	1.82	1.75	1.85	0.88
*monoterpene ketone*																					
camphor	1144	1149	0.00	3.81	1.79	1.20	2	2.16	0.96	3.12	0.40	0.80	0.58	1.07	1.02	0.00	1.83	1.12	1.75	1.30	0.85
*monoterpene ketone*																					
camphene hydrate	1156	1152	0.00	3.41	1.51	0.94	2	1.38	0.45	2.58	0.47	0.95	0.59	1.11	1.11	0.00	1.46	0.81	1.55	1.07	0.79
*monoterpene alcohol*																					
**borneol**	1169	1173	0.48	11.67	4.50	2.54	0	4.09	1.48	6.47	2.15	3.34	1.38	4.11	3.67	0.07	4.30	2.11	4.70	2.97	0.67
*monoterpene alcohol*																					
bornyl acetate	1282	1285	0.00	32.19	9.84	6.93	1	9.40	3.05	17.64	8.54	4.19	2.60	8.14	3.75	0.00	8.15	4.63	11.53	8.46	0.17
*monoterpene ester*																					
(E)-caryophyllene	1418	1424	0.00	9.97	2.64	2.11	1	2.92	2.17	2.18	1.83	3.24	3.12	2.23	1.00	0.71	3.18	2.39	2.11	1.70	0.16
*sesquiterpene hydrocarbon*																					
**α-humulene**	1453	1454	0.22	8.82	1.94	2.00	0	1.94	1.90	0.76	0.60	3.38	2.97	1.69	0.91	0.06	1.76	1.53	2.12	2.42	0.62
*sesquiterpene hydrocarbon*																					
δ-cadinene	1516	1518	0.00	6.25	2.03	1.75	4	2.60	1.67	0.34	0.27	2.16	1.86	3.01	1.57	0.01	1.82	1.69	2.23	1.84	0.52
*sesquiterpene hydrocarbon*																					
**T-muurolol**	1642	1645	0.16	10.58	2.38	1.99	0	1.77	1.23	1.16	1.08	3.45	3.09	3.15	1.06	0.05	2.36	2.60	2.40	1.17	0.96
*sesquiterpene alcohol*																					
α-cadinol	1653	1659	0.00	16.56	6.41	4.87	3	5.46	3.83	2.44	1.91	7.18	5.32	10.55	4.38	0.01	5.85	5.35	6.96	4.45	0.53
*sesquiterpene alcohol*																					
manool	2050	2062	0.00	40.88	10.96	11.83	4	10.21	9.49	1.51	1.59	18.73	11.43	13.38	14.76	0.02	11.88	12.94	10.03	10.95	0.67
*diterpene alcohol*																					
**abienol**	2141	2152	0.19	21.48	3.83	4.29	0	1.13	0.89	2.32	1.92	8.44	6.08	3.44	2.20	0.00	4.39	5.41	3.27	2.87	0.47
*diterpene alcohol*																					
								**17 November**		**18 April**		**18 June**		**18 August**			**Branch**		**Annual shoots**		
**Monoterpene hydrocarbons**			22.89					33.17		37.75		4.03		16.63			25.95		19.83		
**Oxygenated monoterpenes (alcohols, esters, ketones)**			19.34					18.89		33.71		9.43		15.31			17.39		21.28		
**Sesquiterpene hydrocarbons**			6.61					7.46		3.28		8.78		6.93			6.76		6.46		
**Oxygenated sesquiterpenes (alcohols, esters, ketones)**			8.79					7.23		3.6		10.63		13.7			8.21		9.36		
**Diterpene alcohols**			14.79					11.34		3.83		27.17		16.82			16.27		13.3		

**Table 3:** List of volatile compounds whose average abundance according to their relative peak intensity (RPI) (Area %) in 32 hydrosol samples was greater than 1.2% with their log P, minimum, maximum, and average abundance obtained at different season times and with different plant parts with included *p*-value as detected by gas chromatography-mass spectrometry. The compounds were identified based on their mass spectra and retention indices (RI; Db, database RI; Ms, measured RI).

Compound	RI		logP	Minimum RPI (%)	Maximum RPI (%)	Average RPI (%)	Standard Deviation	No. of Samples without Compound	17 November		18 April		18 June		18 August		Seasons	Branch		Annual Shoot		Collection Method
*Chemical Group*	Ms	Db							Average	STD	Average	STD	Average	STD	Average	STD	*p*	Average	STD	Average	STD	*p*
(2E)-2-hexenal	841	850	2.38	0	19.9	2.65	5.25	22	8.7	7	0	0	0	0	1.89	3.79	0	2.92	5.25	2.38	5.40	0.78
*artefact*																						
(3Z)-3-hexen-1-ol	850	853	2.38	0	19.83	5.46	5.80	11	8.78	6.4	0	0	6.58	4.06	6.47	6.51	0.01	4.98	6.43	5.94	5.27	0.65
*artefact*																						
eucalyptol	1030	1032	2.82	0	26.15	8.40	7.80	10	8.27	4.25	17.21	4.84	0.42	1.18	7.7	8.02	0.00	8.14	7.51	8.66	8.31	0.86
*monoterpene ether*																						
camphor	1144	1149	2.13	0	25.04	13.06	6.58	3	13.52	4.54	18.92	4.56	9.02	6.9	10.77	6.21	0.01	14.09	6.73	12.02	6.47	0.38
*monoterpene ketone*																						
camphene hydrate	1152	1156	2.77	2.86	18.11	12.05	4.15	0	9.85	1.93	15.27	1.44	10.99	5.09	12.08	5.1	0.05	12.35	4.54	11.75	3.85	0.69
*monoterpene alcohol*																						
borneol	1169	1173	2.71	0	49.62	27.97	11.85	2	21.65	4.52	29.05	7.33	34.76	11.07	26.43	17.98	0.16	29.86	9.79	26.08	13.67	0.38
*monoterpene alcohol*																						
isoborneol	1169	1165	2.71	0	19.96	1.23	4.85	2	0	0	0	0	0	0	4.93	9.12	0.096	0.00	0.00	2.46	6.73	0.15
*monoterpene alcohol*																						
terpinen-4-ol	1184	1178	2.99	0	40.32	5.55	10.07	6	1.87	0.62	2.52	0.38	11.08	14	6.71	13.78	0.23	6.06	11.88	5.03	8.23	0.78
*monoterpene alcohol*																						
α-terpineol	1193	1195	2.79	0	35.82	12.35	8.53	4	8.36	2.34	12.42	2.15	10.97	11.32	17.63	11.46	0.17	10.87	6.54	13.82	10.14	0.34
*monoterpene alcohol*																						
			Average						17 November		18 April		18 June		18 August		/	Branch		Annual shoots		/
Oxigenated monoterpenes			88.72						81.00		95.39		83.82		94.61		/	89.27		88.14		/

**Figure A1:**
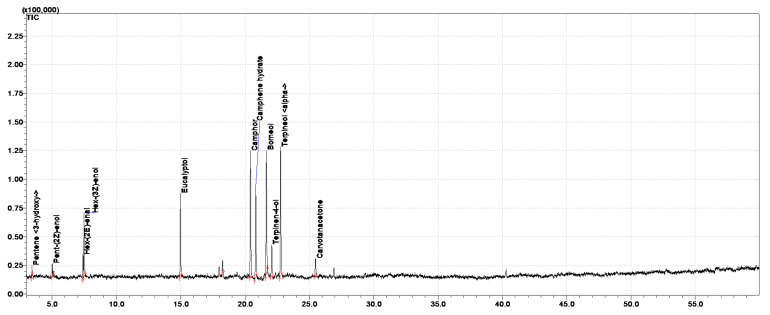
GC-MS chromatogram of hydrosol obtained from *Picea abies* (Bele vode, avg. 2018).

**Figure A2:**
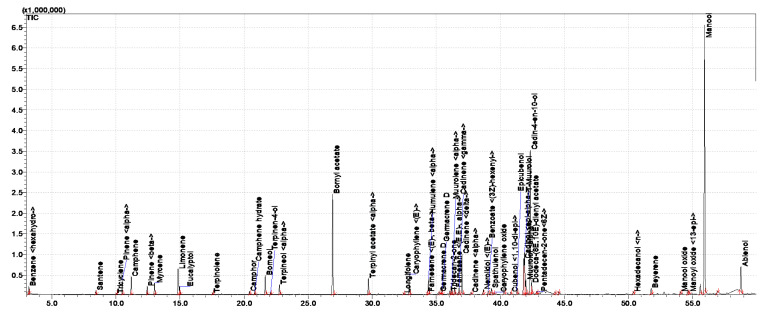
GC-MS chromatogram of essential oil obtained from *Picea abies* (Lazec, avg. 2018).

## Data Availability

Not applicable.

## Appendix A

List of volatile compounds whose average abundance, according to their relative peak intensity (RPI) (Area %), was greater than 0.1% in 32 essential oil samples, with their minimum (Min), maximum (Max), and average abundance as detected by gas chromatography-mass spectrometry. The compounds were identified based on their mass spectra and retention indices (RI; Db, database RI; Ms, measured RI). Unidentified compounds are presented as numbers according to their five most intensive mass ion peaks.

**Compound**	**RI**		**Min**	**Max**	**Average RPI (%)**	**Standard Deviation**
	**Ms**	**Db**				
manool	2050	2062	0.00	40.88	10.96	11.83
bornyl acetate	1282	1285	0.00	32.19	9.84	6.93
limonene	1027	1030	0.19	22.78	6.86	6.00
cadin-4-en-10-ol	1653	1659	0.00	16.56	6.41	4.87
β-pinene	974	978	0.11	28.51	5.59	6.63
borneol	1169	1173	0.48	11.67	4.50	2.54
camphene	946	942	0.15	16.44	3.96	4.19
abienol	2141	2152	0.19	21.48	3.83	4.29
α-pinene	930	933	0.10	9.18	3.25	3.12
myrcene	987	991	0.00	10.95	3.23	3.09
(e)-caryophyllene	1418	1424	0.00	9.97	2.64	2.11
t-muurolol	1642	1645	0.16	10.58	2.38	1.99
delta-cadinene	1516	1518	0.00	6.25	2.03	1.75
α-humulene	1453	1454	0.22	8.82	1.94	2.00
camphor	1144	1149	0.00	3.81	1.79	1.20
eucalyptol	1030	1032	0.00	6.04	1.70	1.81
camphene hydrate	1156	1152	0.00	3.41	1.51	0.94
243 (100) 271 (88) 43 (88) 41 (84) 286 (79)			0.00	43.00	1.43	7.59
α-terpineol	1192	1195	0.34	2.75	1.41	0.65
epi-α-cadinol	1640	1640	0.00	3.64	1.31	1.20
epimanool	2048	2057	0.00	20.45	1.24	4.04
α-bisabolol oxide a	1745	1748	0.00	34.96	1.23	6.20
germacrene d	1473	1480	0.00	4.20	1.01	1.04
longifolene	1407	1412	0.00	4.02	0.86	0.93
(e,e)-α-farnesene	1501	1504	0.00	8.01	0.81	1.68
41 (100) 93 (99) 81 (87) 91 (78) 105 (78)			0.00	12.00	0.80	2.28
α-terpinyl acetate	1344	1349	0.00	2.33	0.77	0.63
43 (100) 81 (79) 41 (70) 93 (66) 107 (64)			0.00	5.14	0.75	1.49
spathulenol	1574	1576	0.00	2.24	0.72	0.59
α-muurolol	1644	1651	0.00	1.48	0.64	0.44
beyerene	1921	1933	0.00	2.99	0.63	0.78
δ-3-carene	1007	1009	0.00	9.04	0.63	1.64
43 (100) 119 (46) 109 (42) 93 (37) 108 (27)			0.00	2.11	0.60	0.60
dodeca-(8e,10e)-dienyl acetate	1655	1665	0.00	3.06	0.55	0.72
caryophyllene oxide	1579	1587	0.00	2.29	0.48	0.56
α-muurolene	1496	1497	0.00	1.23	0.37	0.32
135 (100) 91 (20) 286 (20) 148 (20) 93 (17)			0.00	2.46	0.34	0.64
cyclosativene	1366	1367	0.00	10.54	0.33	1.86
manool oxide	1990	1989	0.00	1.24	0.32	0.36
terpinolene	1083	1086	0.00	1.43	0.31	0.29
santene	879	880	0.00	1.66	0.29	0.36
γ-cadinene	1511	1512	0.00	1.08	0.28	0.28
terpinen-4-ol	1178	1184	0.00	0.62	0.28	0.18
1,2,3,4,4a,7,8,8a-octahydro-4-isopropyl-1,6-dimethyl naphth-1-ol	1638	1641	0.00	2.44	0.27	0.62
(e)-nerolidol	1559	1561	0.00	0.91	0.25	0.22
tricyclene	920	923	0.00	1.27	0.25	0.33
α-bisabolol oxide b	1651	1655	0.00	7.94	0.25	1.40
43 (100) 79 (96) 80 (62) 67 (61) 41 (44)			0.00	1.25	0.24	0.33
273 (100) 105 (62) 148 (48) 43 (46) 107 (45)			0.00	5.00	0.23	0.91
hexahydro-benzene	678	682	0.00	1.49	0.22	0.29
α-bisabolone oxide a	1676	1682	0.00	5.02	0.22	0.90
43 (100) 81 (73) 41 (59) 55 (56) 93 (55)			0.00	2.55	0.21	0.48
abietadiene	2080	2089	0.00	0.95	0.21	0.30
109 (100) 105 (96) 119 (73) 41 (68) 91 (68)			0.00	0.97	0.20	0.26
131 (100) 187 (79) 286 (31) 105 (30) 43 (39)			0.00	2.30	0.19	0.51
humulene epoxide ii	1607	1613	0.00	0.78	0.19	0.22
α-cadinene	1535	1538	0.00	3.00	0.18	0.54
epicubenol	1626	1631	0.00	0.46	0.17	0.15
13-epi-manoyl oxide	2012	2022	0.00	0.75	0.17	0.24
isopulegyl acetate	1269	1273	0.00	5.32	0.17	0.94
43 (100) 79 (90) 80 (62) 67 (56) 41 (44)			0.00	0.71	0.16	0.21
spathulenol	1574	1576	0.00	1.42	0.16	0.43
carvotanacetone	1250	1249	0.00	1.61	0.16	0.38
tridecan-2-one	1491	1495	0.00	0.74	0.15	0.22
(100) 41 (88) 43 (84) 93 (81) 93 (81)			0.00	0.71	0.15	0.17
isopropyl-tetradecanoate	1821	1826	0.00	1.55	0.14	0.34
43 (100) 91 (89) 271 (87) 243 (87) 105 (86)			0.00	2.07	0.14	0.41
phytol	2106	2106	0.00	1.81	0.14	0.38
(e)-β-farnesene	1450	1452	0.00	0.81	0.13	0.20
n-hexadecanol	1877	1884	0.00	2.73	0.12	0.48
γ-terpinene	1056	1058	0.00	0.44	0.12	0.13
cis-muurol-5-en-4-alpha-ol	1573	1558	0.00	3.91	0.12	0.69
6z-pentadecen-2-one	1667	1670	0.00	0.67	0.12	0.20
t-muurolol	1642	1645	0.00	2.99	0.12	0.55
β-phellandrene	1028	1031	0.00	3.35	0.12	0.60
43 (100) 79 (88) 41 (54) 91 (51) 67 (50)			0.00	0.54	0.11	0.16
bicyclogermacrene	1492	1497	0.00	1.01	0.11	0.28
131 (100) 187 (69) 41 (57) 93 (56) 67 (55)			0.00	1.28	0.10	0.25
256 (100) 241 (77) 121 (72) 105 (67) 105 (67)			0.00	1.16	0.10	0.26

## Appendix B

List of volatile compounds whose average abundance, according to their relative peak intensity (RPI) (Area %), was greater than 0.1% in 32 hydrosol samples, with their minimum (Min), maximum (Max), and average abundance as detected by gas chromatography-mass spectrometry. The compounds were identified based on their mass spectra and retention indices (RI; Db, database RI; Ms, measured RI). Unidentified compounds are presented as numbers according to their five most intensive mass ion peaks.

**Compound**	**RI**		**Mini**	**Max**	**Average RPI (%)**	**Standard Deviation**
	**Ms**	**Db**				
borneol	1169	1173	0.00	49.62	27.97	11.85
camphor	1144	1149	0.00	25.04	13.06	6.58
α-terpineol	1193	1195	0.00	35.82	12.35	8.53
camphene hydrate	1152	1156	2.86	18.11	12.05	4.15
eucalyptol	1030	1032	0.00	26.15	8.40	7.80
terpinen-4-ol	1184	1178	0.00	40.32	5.55	10.07
hex-(3z)-enol	850	853	0.00	19.83	5.46	5.80
hex-(2e)-enal	841	850	0.00	19.90	2.65	5.25
isoborneol	1169	1165	0.00	19.96	1.23	4.85
α-bisabolol oxide a	1744	1748	0.00	33.45	1.16	5.93
n-octacosane	2161	2170	0.00	36.07	1.13	6.38
55 (100) 41 (72) 43 (53) 83 (43) 69 (37)			0.00	11.83	1.03	2.67
43 (100) 119 (47) 109 (39) 93 (39) 108 (25)			0.00	8.09	1.00	1.85
bornyl acetate	1282	1285	0.00	6.30	0.92	1.52
carvotanacetone	1250	1249	0.00	11.96	0.91	2.63
3-hydroxy-pentene	689	680	0.00	5.07	0.68	1.33
44 (100) 45 (35) 43 (20) 41 (18) 93 (17)			0.00	12.94	0.59	2.37
hex-(2e)-enal	824	830	0.00	11.09	0.56	2.17
131 (100) 187 (68) 105 (36) 41 (35) 286 (29)			0.00	9.67	0.37	1.73
43 (100) 72 (76) 108 (71) 71 (67) 93 (63)			0.00	3.72	0.34	0.77
pent-(2z)-enol	764	767	0.00	2.44	0.26	0.64
isobornyl acetate	1282	1285	0.00	3.33	0.24	0.71
135 (100) 91 (22) 286 (21) 41 (18) 93 (17)			0.00	4.86	0.19	0.87
43 (100) 243 (93) 271 (76) 41 (74) 91 (69)			0.00	5.39	0.18	0.95
pent-(2e)-enal	748	751	0.00	1.44	0.14	0.36
linalool	1132	1135	0.00	1.71	0.13	0.42
82 (100) 110 (64) 95 (47) 54 (28) 109 (25)			0.00	1.97	0.11	0.39
cis-hex-3-enal	797	797	0.00	1.35	0.10	0.34
77 (100) 45 (20) 78 (7) 59 (6) 62 (6)			0.00	0.87	0.10	0.25

## Appendix C

Sample of GC-MS chromatogram of essential oil and hydrosol obtained from *Picea Abies*.
